# Depression of Vaccinal Immunity to Marek’s Disease by Infection with Chicken Infectious Anemia Virus

**DOI:** 10.3389/fmicb.2017.01863

**Published:** 2017-09-26

**Authors:** Yankun Zhang, Ning Cui, Ni Han, Jiayan Wu, Zhizhong Cui, Shuai Su

**Affiliations:** ^1^College of Veterinary Medicine, Shandong Agricultural University, Tai’an, China; ^2^Shandong Provincial Key Laboratory of Animal Biotechnology and Disease Control and Prevention, Shandong Agricultural University, Tai’an, China; ^3^Shandong Provincial Engineering Technology Research Center of Animal Disease Control and Prevention, Shandong Agricultural University, Tai’an, China; ^4^Institute of Animal Husbandry and Veterinary, Shandong Academy of Agricultural Sciences, Jinan, China

**Keywords:** Marek’s disease virus, infection, chicken infectious anemia virus, depression, vaccinal immunity

## Abstract

Marek’s disease (MD) has been occurring with increasing frequency in chickens in recent years. To our knowledge, however, there has been no report of the very virulent plus (vv+) MD virus (MDV) field isolate in China. Studies have shown that dual infection with immunosuppressive viruses such as chicken infectious anemia virus (CIAV) occurs frequently in chickens developing MD. In this study, we performed a designed set of *in vivo* experiments, which comprised five different groups of chickens, including the group of CVI988/Rispens-vaccinated chickens, the groups of CVI988/Rispens-vaccinated chickens infected with MDV or CIAV or both viruses (MDV and CIAV), and the group of MDV-challenged chickens. The effects of CIAV dual infection on the immunization of commercial MDV vaccine CVI988/Rispens were evaluated. The results show that infection of the SD15 strain of CIAV significantly reduced the weight and antibody titers to avian influenza virus (AIV)/Newcastle disease virus (NDV) inactivated vaccines of chickens immunized with the CVI988/Rispens, and resulted in the atrophy of thymus/bursa and the enlargement of spleen. The CVI988/Rispens vaccination conferred good immune protection for chickens challenged with 2000 PFU of the GX0101 strain of MDV. However, dual infection with SD15 significantly reduced the body weight, antibody titers induced by AIV/NDV inactivated vaccines and protective index of CVI988/Rispens, and resulted in the aggravation of the immunosuppression, mortality, and viremia of GX0101 in CVI988/Rispens-immunized/GX0101-challenged chickens. Overall, CIAV infection significantly reduced the protective effects of the CVI988/Rispens vaccine against MDV, implying that concurrent infection with CIAV may be a major contributor in the frequent attacks of MD in China in recent years.

## Introduction

Marek’s disease (MD) is a lymphoproliferative disease of chickens, which is caused by the MD virus (MDV) (Schat and Nair, 2008). MDVs are further divided into pathotypes, ranging from mild (m), virulent (v), and very virulent (vv) to very virulent plus (vv+) strains (Witter, 1997; Witter et al., 2005). MD is currently the only tumor disease in chickens that can be immunized against by vaccine. After the first case of MD in 1960, HPRS-16/ATT (HPRS, Houghton Poultry Research Station), herpesvirus of turkeys (HVT), and HVT plus SB-1 or 301B/1 were developed to control MD (Churchill et al., 1969a,b; Okazaki et al., 1970; Witter et al., 1987). In the 1990s, CVI988/Rispens became the worldwide vaccine gold standard (Rispens et al., 1972). Recently, the “gold-standard” vaccine CVI988/Rispens has gradually showed poor protective efficacy against MDV in China (Teng et al., 2011; Tian et al., 2011; Cheng et al., 2012; Yu et al., 2013; Zhang et al., 2015; Cui et al., 2016). Several factors including the genetic background of chickens, the virulence of MDV, and concurrent infections with other immunosuppressive pathogens can influence the efficacy of MDV vaccines (Bacon et al., 2001). Although the use of vaccines may lead to an enhanced virulent strain of MDV, there has been no report of the vv+ MDV field isolate in China.

Concurrent infection with other viruses is very common in chickens with MD. This is particularly true of immunosuppressive viruses such as chicken infectious anemia virus (CIAV), avian reticuloendotheliosis virus (REV), and avian leukosis virus (ALV) (Qin et al., 2010; Zhao et al., 2012; Cui, 2013; Bao et al., 2015; Ahmed et al., 2016). CIA, which caused by CIAV, is characterized by aplastic anemia and immunosuppression in chickens (Miller and Schat, 2004). Chickens can be infected with CIAV, both vertically and horizontally (Hoop, 1992). CIAV is increasing in prevalence and infection increases susceptibility to a wide variety of other avian pathogens, presumably through immunosuppression of the CIAV-infected bird (Todd, 2004). Dual infection with CIAV and MDV showed synergistic effects on the pathogenicity with enhanced mortality and incidence of MD (Yang et al., 2010). Therefore, concurrent infection with CIAV is likely to be a factor in the increasingly frequent occurrences of MD in China in recent years. In this study, we analyzed the effects of CIAV dual infection on the immunization of commercial MDV vaccine CVI988/Rispens to better facilitate the establishment of effective control measures for MD in chickens.

## Materials and Methods

### Ethics Statement

The study protocol and all animal studies were approved by the Shandong Agricultural University Animal Care and Use Committee (SACUC Permission number: AVM201701-2) and performed in accordance with the “Guidelines for Experimental Animals” of the Ministry of Science and Technology (Beijing, China). Any bird deemed to have reached the humane endpoint was culled.

### Cell Culture and Viruses

Specific pathogen-free (SPF) chickens and chicken embryos used for the preparation of chicken embryo fibroblast (CEF) cultures were from SPAFAS Co. (Jinan, China). GX0101 strain of vv MDV and SD15 strain of CIAV were preserved in our laboratory (Zhang and Cui, 2005; Fang, 2017). MDV vaccine CVI988/Rispens was purchased from Merial Animal Health Co., Ltd.

### Experimental Design

The experimental plan was illustrated in **Supplementary Figure S1**. Two-hundred SPF chickens were randomly divided into five equal groups (40 in each group) at 1 day old and reared separately in isolators with positive filtered air. All chickens of groups 1, 2, 3, and 4 were intra-abdominally (i.a.) infected at 1 day old with CVI988/Rispens. Groups 2 and 3 were inoculated intra-oral in addition with 400 EID_50_ of SD15 (Fang, 2017). Five days later, each chicken in groups 3, 4, and 5 was challenged i.a. with 2000 PFU of GX0101.

### Measurement of Body Weight and Immune Organs Indices

The body weight of the chickens in different groups was measured at 0, 5, 9, 16, 23, 30, 37, and 44 days post-infection (dpi) with GX0101 to evaluate the effect of viral infection on growth rates. After 9 and 16 dpi, five chickens per group were used to evaluate the immune organs indices. The whole-body weight of each chicken was measured prior to euthanasia, and the thymus, spleen, and bursa from each chicken were collected and weighed. The immune organs indices were determined by the relative weight of the thymus, spleen, and bursa to the whole body.

### Antibody Responses to Newcastle Disease Virus (NDV) and Avian Influenza Virus (AIV)–H9 Inactivated Vaccines

All chickens from each treatment group were vaccinated with Newcastle Disease Virus (NDV) and Avian Influenza Virus (AIV)–H9 inactivated vaccines according to the previously described procedure at 8 days old (Sun et al., 2007). On days 21, 28, and 35 post-vaccination, serum samples were randomly collected from chickens of each group. Hemagglutination inhibition (HI) antibody titers against NDV and AIV–H9 were determined in accordance with the routine procedures.

### Protective Efficacy of CVI988/Rispens Vaccine

During 90 days post-challenges with GX0101, each dead chicken was recorded and necropsied. At the end of the study period, all surviving chickens were euthanized for autopsy. The protective efficacy of the vaccine for MD was expressed as a protective index (PI) calculated as the percentage of gross MD in non-vaccinated challenged control chickens minus the percentage of gross MD in vaccinated, challenged chickens divided by the percentage of gross MD in non-vaccinated challenged control chickens × 100.

### Quantification of Viral Load

Blood samples in anticoagulants were collected from six chickens of each of the GX0101-infected groups (groups 3, 4, and 5) at 5, 9, 16, 23, and 30 dpi. DNA from peripheral blood lymphocytes (PBLs) were extracted using standard procedures (Sambrook et al., 1989). The MDV-specific primers were designed to be specific for the unique molecular marker of REV LTR in GX0101 (Duan et al., 2014). GX0101 DNA in PBLs was quantified with real-time quantitative PCR (RT-qPCR) according to the previous method (Duan et al., 2014). qPCR reactions were set up on ice, and each reaction contained the following: MDV-specific primers (all at 0.5 uM), 10 ul SYBR Premix Ex Taq^TM^ (2×), 0.4 ul Rox Reference Dye II (50×), and 2 ul of DNA (approximately 100 ng). The reaction volume was brought up to 20 ul by the addition of ddH_2_O. An ABI PRISM^®^ 7500 Sequence Detection System (Applied Biosystems) was used to amplify and detect the reaction products.

### Quantification of Cytokine mRNA Expression

Total RNA was extracted from PBLs collected from six chickens of each group (groups 3 and 4) at 0, 5, 9, 16, and 23 dpi with GX0101. The production of cytokine mRNA of interleukin-6 (IL-6), IL-18, and gamma interferon (IFN-γ) at different stages was quantified by RT-quantitative reverse transcription PCR (qPCR) according to the previous method (Jie et al., 2013; Heidari et al., 2016). Briefly, 2 μl of the oligo dT-based RT product from 4 μg of total RNA extracted from PBLs was used for each reaction. All the reactions were run in triplicates in an ABI PRISM^®^ 7500 Sequence Detection System (Applied Biosystems). The amplification program was as follows: 95°C for 30 s, 40 cycles at 95°C for 5 s, 60°C for 34 s, followed by 95°C for 15 s, 60°C for 1 min, and 95°C for 15 s. The relative expression ratios of target genes in the chickens of group 3 vs. those in group 4 were calculated by the 2^-ΔΔCt^ method using the chicken housekeeping gene β-actin as the endogenous reference gene in order to normalize the level of target gene expression.

### Statistical Analysis

Statistical analysis was performed with the SPSS statistical software package for Windows, version 13.0 (SPSS Inc., Chicago, IL, United States). Differences between groups were examined for statistical significance by a two-tailed Student’s *t*-test. A *p*-value <0.05 was considered statistically significant. Pairwise comparisons of the PI between vaccines were approximated using *Z*-statistic for difference between proportion data with Bonferroni corrections (Geng and Hills, 1989).

## Results

### Body Weights

**FIGURE 1:**
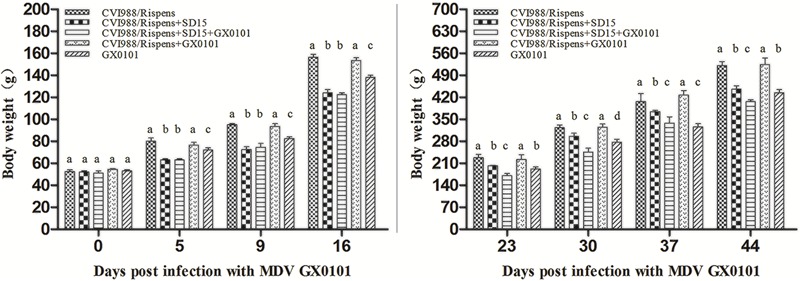
The body weights of chickens in each group. The body weight of the chickens in different groups was measured at 0, 5, 9, 16, 23, 30, 37, and 44 days post-infection with GX0101 to evaluate the effect of virus infection on growth rates. ^a,b,c,d^The different letters represent significant differences (*p* < 0.05). The same letters indicate the differences were not significant (*p* > 0.05).

No significant differences were observed between different groups in the body weight of 6 days old chickens (*p* > 0.05) (**Figure 1**). At 5, 9, 16, 23, 30, 37, and 44 dpi with GX0101, there was no significant difference in body weight between group 1 and group 4 (*p* > 0.05), while that of the chickens in group 4 was significantly higher than those in group 5 (*p* < 0.05), indicating that CVI988/Rispens could prevent weight loss caused by GX0101 infection in SPF chickens. The body weight of chickens in group 1 was significantly increased as compared to that of the group 2 (*p* < 0.05), and the body weight of chickens in group 3 was significantly decreased as compared to that of group 4 (*p* < 0.05), suggesting that the body weight of chickens vaccinated with CVI988/Rispens, especially that of the CVI988/Rispens-vaccinated/GX0101-challenged chickens, was reduced by SD15 infection.

### Immune Organs Indices

Chickens in group 5 exhibited an atrophied thymus and bursa of Fabricius with an enlarged spleen as compared to that of the chickens from group 1 (*p* < 0.05) after 9 and 16 dpi with GX0101 (**Table 1**). No significant change was observed in the chickens of group 4 (*p* > 0.05) with the exception of spleen enlargement presenting in chickens challenged with GX0101 at 9 dpi, indicating that CVI988/Rispens could reduce the damage of GX0101 to the immune organs in SPF chickens. Atrophy of thymus and bursa of Fabricius as well as spleen enlargement were noted in group 2 as compared to that of the chickens in group 1 (*p* < 0.05). Chickens in group 3 showed an atrophied thymus and bursa of Fabricius and enlarged spleen as compared to those of the chickens from group 4 (*p* < 0.05). These results demonstrated that SD15 infection significantly reduced the protective efficacy of CVI988/Rispens on immune organs in immunized chickens, especially those in the CVI988/Rispens-vaccinated/GX0101-challenged group (group 3).

**Table 1:** The results of relative immune organs weight (*n* = 5).

Virus	Immune organs indices (9 dpi with GX0101)			Immune organs indices (16 dpi with GX0101)		
	Thy^∗^ (%)	Spl (%)	Bur (%)	Thy (%)	Spl (%)	Bur (%)
CVI988/Rispens	0.438 ± 0.037^a^	0.150 ± 0.020^a^	0.188 ± 0.034^a^	0.533 ± 0.064^a^	0.221 ± 0.017^a^	0.395 ± 0.011^a^
CVI988/Rispens+SD15	0.188 ± 0.012^b^	0.241 ± 0.046^b^	0.129 ± 0.049^b^	0.240 ± 0.016^b^	0.443 ± 0.032^b^	0.299 ± 0.064^b^
CVI988/Rispens+SD15+GX0101	0.177 ± 0.022^b^	0.293 ± 0.023^c^	0.107 ± 0.033^b^	0.124 ± 0.022^c^	0.689 ± 0.014^c^	0.208 ± 0.054^c^
CVI988/Rispens+GX0101	0.392 ± 0.040^a^	0.198 ± 0.021^d^	0.178 ± 0.021^a^	0.503 ± 0.030^a^	0.244 ± 0.041^a^	0.386 ± 0.041^a^
GX0101	0.254 ± 0.007^c^	0.336 ± 0.023^e^	0.138 ± 0.022^b^	0.254 ± 0.007^b^	0.488 ± 0.041^b^	0.296 ± 0.052^b^

*The numbers in the table indicate the mean ± standard deviation. dpi, days post-infection. ^a,b,c,d,e^The different letters represent significant differences (*p* < 0.05). The same letters indicate the differences were not significant (*p* > 0.05). ^∗^Thy, relative thymus weight; Bur, relative bursa weight; Spl, relative spleen weight.*

### Antibody Titers to AIV–H9 and NDV of Chickens in Different Groups

On 21, 28, and 35 days post-immunization with the inactivated vaccines, antibody titers to AIV–H9 and NDV in chickens from group 2 were significantly lower than that of the chickens from group 1, respectively (*p* < 0.05) (**Table 2**). Antibody titers to AIV–H9 of chickens from groups 3 and 5 were significantly decreased, and antibody titers to NDV were significantly decreased at 35 days post-immunization as compared to those of the chickens from group 4 (*p* < 0.05). The results indicated that SD15 led to immunosuppressive effects on humoral immune responses in the CVI988/Rispens-vaccinated chickens, especially on that of the CVI988/Rispens-vaccinated/GX0101-challenged chickens (group 3).

**Table 2:** The antibody response to vaccination with NDV and AIV–H9 inactivated vaccines.

Virus	HI antibody titers to AIV–H9			HI antibody titers to NDV		
	21 days	28 days	35 days	21 days	28 days	35 days
CVI988/Rispens	6.3 ± 0.89 (30)^a^	7.3 ± 0.99 (30)^a^	7.4 ± 0.93 (30)^a^	7.1 ± 0.35 (30)^a^	7.5 ± 0.77 (30)^a^	7.8 ± 0.90 (30)^a^
CVI988/Rispens+SD15	5.3 ± 0.46 (25)^b^	5.9 ± 0.64 (25)^b^	6.3 ± 0.46 (25)^b^	5.8 ± 0.89 (25)^b^	6.1 ± 0.52 (25)^b^	6.9 ± 0.64 (24)^b^
CVI988/Rispens+SD15+GX0101	4.6 ± 0.95 (17)^c^	4.3 ± 1.17 (16)^c^	4.8 ± 0.42 (15)^c^	5.6 ± 0.79 (17)^b^	6.0 ± 0.65 (16)^b^	5.8 ± 0.71 (15)^c^
CVI988/Rispens+GX0101	5.9 ± 0.99 (30)^ab^	6.9 ± 0.64 (29)^a^	7.6 ± 0.92 (29)^a^	6.4 ± 0.52 (30)^b^	6.4 ± 0.71 (29)^b^	7.5 ± 0.52 (29)^a^
GX0101	4.3 ± 0.71 (24)^c^	5.3 ± 0.89 (23)^b^	5.6 ± 0.87 (21)^b^	6.0 ± 0.87 (24)^b^	6.4 ± 0.92 (23)^b^	6.9 ± 0.88 (21)^b^

*The numbers in the table indicate the mean ± standard deviation (sample size). ^a,b,c^The different letters represent significant differences (*p* < 0.05). The same letters indicate the differences were not significant (*p* > 0.05).*

### Protective Efficacy of CVI988/Rispens Vaccination Against Challenge of GX0101 in SPF Chickens

**FIGURE 2:**
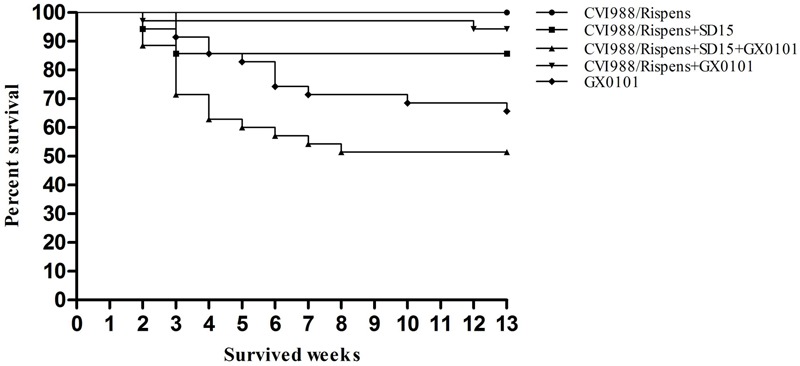
Incidence of mortality in chickens inoculated with MDV GX0101. Chickens were inoculated with 2000 PFU of MDV GX0101 when they were 6 days old and were maintained in isolation for 13 weeks. During the experiment, all dead chickens were recorded and necropsied.

During the entire trial, chickens grew well, and no chickens died in the CVI988/Rispens-vaccinated group (**Figure 2**). The mortality rates of groups 2, 3, 4, and 5 were 14.3, 42.9, 5.7, and 31.4%, respectively (**Table 3**). In the GX0101-challenged groups, one chicken in group 3 and three chickens in group 5 developed visible tumor nodules, but no chicken developed visible MDV-induced lesion in group 4. CVI988/Rispens protected 94.3% of the chickens in group 4 while only protecting 54.3% of the chickens in group 3. These results indicate that the dual infection of SD15 significantly increased the GX0101-induced mortality rate and decreased the protective efficacy of the CVI988/Rispens vaccination.

**Table 3:** Protective efficacy of CVI988/Rispens against challenge of vv MDV GX0101 in SPF chickens.

Virus	Lesions	Mortality	Tumors rate	PI
CVI988/Rispens	–	–	–	–
CVI988/Rispens+SD15	14.3%	14.3%	0%	–
CVI988/Rispens+SD15+GX0101	45.7%	42.9%	2.9%	54.3%^a^
CVI988/Rispens+GX0101	5.7%	5.7%	0%	94.3%^b^
GX0101	100%	31.4%	8.6%	–

*^a,b^The different letters represent significant differences (*p* < 0.05).*

### Replication of GX0101 in SPF Chickens

**FIGURE 3:**
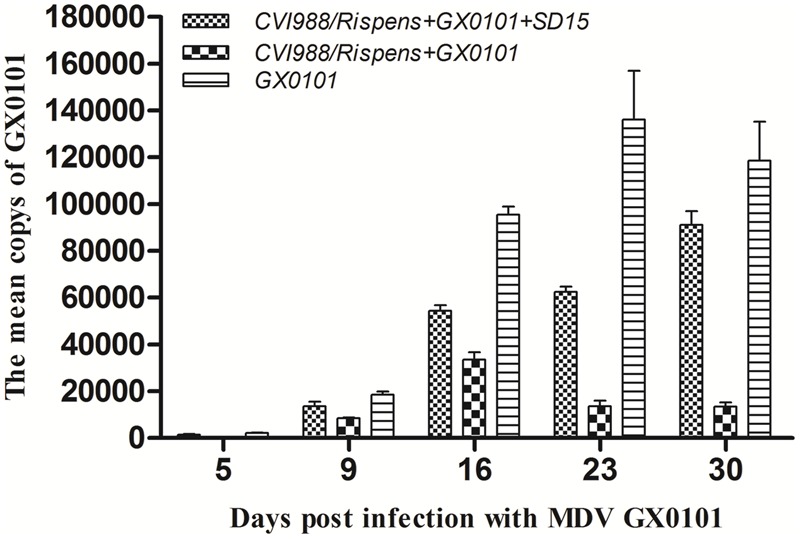
Replication kinetics of MDV GX0101 in chickens. Replication kinetics of GX0101 viruses *in vivo* as determined by the viral genome copy numbers in the PBLs with real-time qPCR of the REV LTR fragment.

Replication of MDV in the chickens of group 5 peaked at 23 days post-challenge with GX0101, while that in the chickens of group 4 peaked at 16 dpi, with a significantly lower MDV copy number than that of the group 5 (*p* < 0.05) (**Figure 3**). This indicates that the CVI988/Rispens vaccine could significantly reduce the replication of GX0101. GX0101 increased continuously in chickens of group 3 and reached its peak at 30 dpi, with a significantly higher virus copy number than that of the group 4 (*p* < 0.05).

### Cytokine mRNA Expression Levels

**FIGURE 4:**
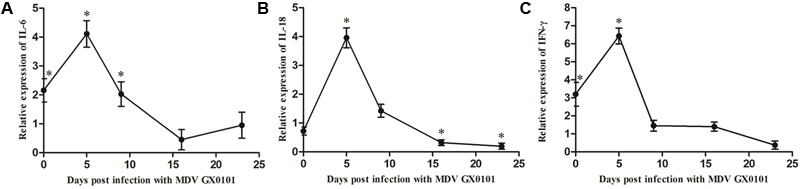
Cytokine mRNA expression level in PBLs of the chickens. The relative expression levels of **(A)** IL-6, **(B)** IL-18, and **(C)** INF-γ genes in the chickens of group 3 vs. those in group 4 were determined by RT-qPCR. The chickens in group 3 were both vaccinated with CVI988/Rispens and infected with SD15 at 1 day old, and challenged with GX0101 at 5 days later. The chickens in group 4 were vaccinated with CVI988/Rispens at 1 day old and challenged with GX0101 at 5 days later. ^∗^Indicates significant difference (*p* < 0.05) between the two experimental groups.

The expression of mRNA for IL-6 and INF-γ increased in chickens from group 3 while there was no significant difference in the expression of mRNA for IL-18 as compared with the values for group 4 in 6 days old chickens (**Figure 4**). The expressions of mRNA for IL-6, IL-18, and INF-γ increased significantly at 5 dpi in chickens from group 3, and then decreased to a level significantly lower than those of group 4 until 16 dpi.

## Discussion

Marek’s disease infection has occurred with increasing frequency in chickens in recent years, but there has been no report concerning the isolation of the vv+ MDV field strain in China (Teng et al., 2011; Tian et al., 2011; Cheng et al., 2012; Yu et al., 2013; Zhang et al., 2015; Cui et al., 2016). China is rich in genetic resources related to chickens, and various species of indigenous breeds scattered throughout the country. Long-term mixed breeding led to the dissemination of different viruses among chickens, especially CIAV, ALV, and REV (Qin et al., 2010; Zhao et al., 2012; Bao et al., 2015). The sub-clinical disease of commercial broilers due to CIAV is more common than clinical disease (McNulty, 1991). In chickens with an outbreak of MD, dual infection with MDV and other immunosuppressive viruses (and even triple infection) were detected (Cui, 2013). In the current study, we systematically evaluated the influence of CIAV infection on the immune efficacy of CVI988/Rispens in chickens.

Our study shows that the dual infection of SD15 significantly reduced the body weight of chickens immunized with CVI988/Rispens and induced severe thymus/bursa atrophy and immunosuppression with significantly inhibited production of antibodies to AIV/H9 and NDV inactivated vaccines (**Tables 1, 2** and **Figure 1**). A vaccinated model was then established using MDV-infected SPF chickens. The vv MDV GX0101 strain used for challenge is a recombinant field MDV that contains a REV LTR fragment (Cui et al., 2010; Su et al., 2012). The REV LTR was then selected as a molecular marker to differentiate CVI988/Rispens and to detect the multiplication level of GX0101. Our research demonstrates that CVI988/Ripens could provide good immunoprotection against challenge with 2000 PFU of GX0101 in SPF chickens at 6 days of age (**Table 3** and **Figure 2**). Replication of GX0101 as well as its pathogenicity in infected chickens was effectively decreased by CVI988/Rispens vaccination (**Table 3** and **Figures 2, 3**). However, dual infection of SD15 significantly reduced the body weight and the antibody titers to AIV/NDV-inactivated vaccines in CVI988/Rispens-immunized/GX0101-challenged chickens while increasing the immunosuppression and mortality (**Tables 1, 2** and **Figure 1**). The PI of CVI988/Rispens against GX0101 challenge was also significantly decreased with increased viral titers of GX0101 in SPF chickens (**Table 3** and **Figure 3**). MDV vaccine has a protective effect in chickens but does not entirely prevent infection nor the replication of virulent virus. Our research and previous studies consistently demonstrated that the MDV vaccine with good immunogenicity could effectively inhibit the replication of wild strains of MDV. However, dual infection of CIAV poses a serious threat to the commercial CVI988/Rispens vaccine, causing considerable replication and long-term excreting of MDV in immunized chickens, which resulted in the enhanced transmission of MDV among chickens. Under the immune selective pressure, the virulence of field MDV showed a gradually increasing trend (Gimeno, 2008; Davison and Nair, 2014).

Cytokines play a critical role in driving immune response to MDV (Kaiser et al., 2003). Expressions of mRNA for IL-6 and INF-γ were increased significantly due to dual infection with CIAV in the chickens of the CVI988/Rispens group at 6 days old (**Figure 4**). Preliminary studies reported that IL-6 and IFN-γ mRNA transcript levels increased during early stages of infection with CIAV (Giotis et al., 2015). Expressions of mRNA for IL-6, IL-18, and INF-γ increased significantly and then decreased after 5 dpi with GX0101 in chickens of the CVI988/Rispens-vaccinated/SD15-inoculated group. IFN-γ plays a pivotal role in the early pathogenesis and immune responses to MDV infection (Xing and Schat, 2000; Abdul-Careem et al., 2007). It has been considered to be an immuno-modulator and vaccine adjuvant against MDV. Expression of recombinant chicken IFN-γ in HVT enhanced the protective efficacy of the vaccine against MDV and reduced the viral load and tumor incidence (Haq et al., 2011). IL-18 is a proinflammatory cytokine that induces IFN-γ production from CD4+T cells (Gobel et al., 2003). Thus, the reduced level of mRNA for IL-18 and IFN-γ in the late stage of infection probably correlates to the decline in the protective efficacy of the MDV vaccine. IL-6 is also a proinflammatory cytokine and its function in MDV infection is still unclear. The potential role for IL-6 in the immune response to MDV has been shown by a mouse model for another α-herpesvirus, herpes simplex virus-1. Mice showing an IL-6 deficiency when infected with HSV-1 have been shown to have increased viral titers and high mortality rates (Murphy et al., 2008). A similar IL-6 deficiency might also contribute to the increased titer of the MDV field strain and the depression of vaccinal immunity of the MD vaccine in chickens co-infected with CIAV.

## Conclusion

Chickens concurrently infected with CIAV showed a declined immune efficacy of CVI988/Rispens against MD and a significantly enhanced susceptibility to MDV. Thus, CIAV might be a factor in frequent attacks of MD in chickens. In order to enhance the prevention and control of MD in chickens, detection of CIAV in chickens should be emphasized. However, no better measures are available for the control of CIAV (Cui, 2015). Most importantly, it is imperative that new vaccination strategies should be developed in case the currently available vaccines lose efficacy in controlling MDV strains with greater virulence (Lee et al., 2008; Su et al., 2015). Development of a recombinant MDV vector vaccine against CIAV is also a desirable application (Moeini et al., 2011; Reddy et al., 2016).

## Author Contributions

YZ collected and assembled, the data, did manuscript writing, and data analysis; NC and SS discussion, manuscript revision; NH and JW performed the animal experiments; SS and ZC concept and design, data analysis, manuscript revision, and final approval of the manuscript.

## Conflict of Interest Statement

The authors declare that the research was conducted in the absence of any commercial or financial relationships that could be construed as a potential conflict of interest. The reviewer RR and handling Editor declared their shared affiliation.

## Supplementary Material

FIGURE S1 Flow chart of the experimental design.
