# Supplementary figures and images for: Depression of Vaccinal Immunity to Marek’s Disease by Infection with Chicken Infectious Anemia Virus
Source: Front Microbiol. 2017 Sep 26;8:1863. doi: 10.3389/fmicb.2017.01863 (PMC5622928; doi:10.3389/fmicb.2017.01863)

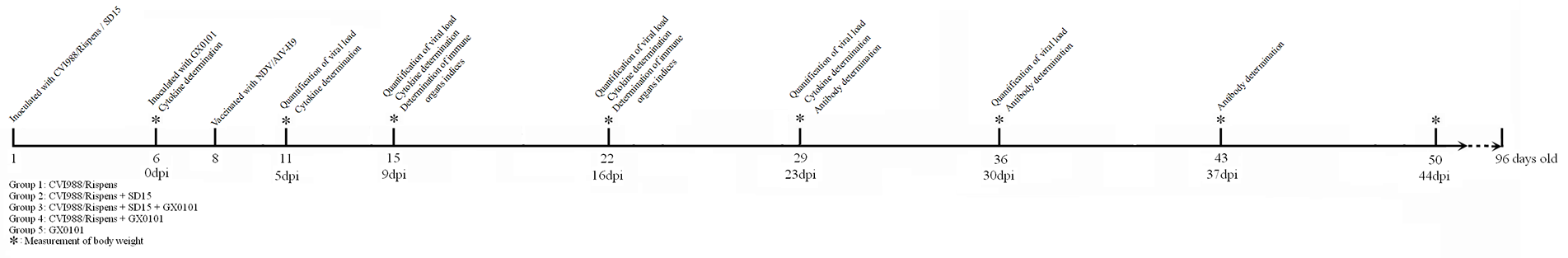

Supplement: FIGURE S1 — Flow chart of the experimental design. [file Image_1.TIF]
